# Genome-wide epistasis analysis for Alzheimer’s disease and implications for genetic risk prediction

**DOI:** 10.1186/s13195-021-00794-8

**Published:** 2021-03-04

**Authors:** Hui Wang, David A. Bennett, Philip L. De Jager, Qing-Ye Zhang, Hong-Yu Zhang

**Affiliations:** 1 Huazhong Agricultural University, College of Informatics, Hubei Key Laboratory of Agricultural Bioinformatics, No.1 Shizishan Street, Hongshan District, Wuhan, 430070 Hubei China; 2 Rush University Medical Center, Rush Alzheimer’s Disease Center, Chicago, IL USA; 3 Rush University Medical Center, Department of Neurological Sciences, Chicago, IL USA; 4 Columbia University Medical Center, Center for Translational and Computational Neuroimmunology, New York, NY USA; 5 Broad Institute, Cell Circuits Program, Cambridge, MA USA

**Keywords:** Alzheimer’s disease, Association studies in genetics, Gene expression studies, Polygenic risk score

## Abstract

**Background:**

Single-nucleotide polymorphisms (SNPs) identified by genome-wide association studies only explain part of the heritability of Alzheimer’s disease (AD). Epistasis has been considered as one of the main causes of “missing heritability” in AD.

**Methods:**

We performed genome-wide epistasis screening (*N* = 10,389) for the clinical diagnosis of AD using three popularly adopted methods. Subsequent analyses were performed to eliminate spurious associations caused by possible confounding factors. Then, candidate genetic interactions were examined for their co-expression in the brains of AD patients and analyzed for their association with intermediate AD phenotypes. Moreover, a new approach was developed to compile the epistasis risk factors into an epistasis risk score (ERS) based on multifactor dimensional reduction. Two independent datasets were used to evaluate the feasibility of ERSs in AD risk prediction.

**Results:**

We identified 2 candidate genetic interactions with *P*_*FDR*_ <  0.05 (*RAMP3*-*SEMA3A* and *NSMCE1*-*DGKE*/*C17orf67*) and another 5 genetic interactions with *P*_*FDR*_ <  0.1. Co-expression between the identified interactions supported the existence of possible biological interactions underlying the observed statistical significance. Further association of candidate interactions with intermediate phenotypes helps explain the mechanisms of neuropathological alterations involved in AD. Importantly, we found that ERSs can identify high-risk individuals showing earlier onset of AD. Combined risk scores of SNPs and SNP-SNP interactions showed slightly but steadily increased AUC in predicting the clinical status of AD.

**Conclusions:**

In summary, we performed a genome-wide epistasis analysis to identify novel genetic interactions potentially implicated in AD. We found that ERS can serve as an indicator of the genetic risk of AD.

**Supplementary Information:**

The online version contains supplementary material available at 10.1186/s13195-021-00794-8.

## Background

Alzheimer’s disease (AD) is a chronic neurodegenerative disease that is characterized by the extracellular deposition of beta-amyloid and the intracellular accumulation of phosphorylated tau protein. AD is the most common cause of dementia in elderly people, with an incidence rate of approximately 1.5% among people over 65 years old and nearly 50% among people over 90 years old [1]. Unlike early-onset AD, which is often caused by mutations in *APP*, *PSEN1*, or *PSEN2* [2], late-onset AD (LOAD), the most common form of AD, exhibits a more complex genetic mechanism. Apolipoprotein ε4 allele (*APOE4)* is the only common high-risk genetic variant associated with LOAD, and previous large-scale genome-wide association studies (GWASs) have identified dozens of loci with small effects [3–5], suggesting that a large portion of the genetic components of LOAD remains unexplained.

It is estimated that 24–33% of the phenotypic variance of LOAD can be explained by *APOE* combined with common variants [6, 7]; this value is considerably lower than the well-reported 58–79% of heritability estimated from twin studies [8]. Rare variants, structural variants, and genetic interactions are possible causes of missing heritability in complex diseases [9]. Previous studies have identified rare coding variants in *SORL1* and *ABCA7*, which can affect APP processing [10, 11]. Besides, rare variants in *PLCG2*, *ABI3*, and *TREM2* revealed the involvement of microglial-mediated innate immunity in AD [12]. In this study, we focus on the genetic interactions, which refers to the combinatorial effect of one or more variants, to help explain the missing heritability in AD. However, there are several challenges in detecting genetic interactions on a genome-wide scale. First, the computational burden of testing pairwise interactions exhaustively is heavy due to the quadratic complexity involved [13]. While a typical GWAS analysis analyzes several million SNPs, corresponding genome-wide interaction screening needs to be performed on more than 1 × 10^14^ SNP interactions, which is a prohibitive number. Second, the detection of genetic interactions is a typical case of a “large p, small n” problem [14]. To reduce the high rate of false positives caused by the astronomic number of tests performed, *P* value thresholds tend to be extremely conservative, while the sample size is usually the same as in traditional GWAS analysis, which can lead to a failure in discovering significant genetic interactions. Finally, the biological interpretation of statistical interactions is challenging, as statistical interactions do not necessarily imply an interaction at the biological level [15]. This situation is further complicated by the problem of insufficient sample size, as samples are stratified into the 9 cells of a 3 × 3 contingency table instead of the 3 groups discriminated by the counts of minor alleles in a typical GWAS analysis. The small sample size in the cells of the 3 × 3 contingency table could lead to invalid biological interpretations of statistical interactions. Due to these limitations, only one genome-wide interaction analysis has identified an interaction between rs6455128 (*KHDRBS2*) and rs7989332 (*CRYL1*) that is replicable across datasets [16]. In this study, we limited the analysis to SNPs that are more likely to be deleterious according to combined annotation-dependent depletion (CADD) scoring. Therefore, the number of tests to be performed was scaled down. The aforementioned first and second problems of genetic interaction screening were alleviated. For better biological interpretation of statistical interactions, we excluded interactions with any of the cells containing less than 3 samples in the 3 × 3 × 2 contingency table. Furthermore, we analyzed the associations of candidate interactions with intermediate AD pathologies, including brain atrophy, white matter injury, and tau and amyloid deposition.

**Fig. 1:**
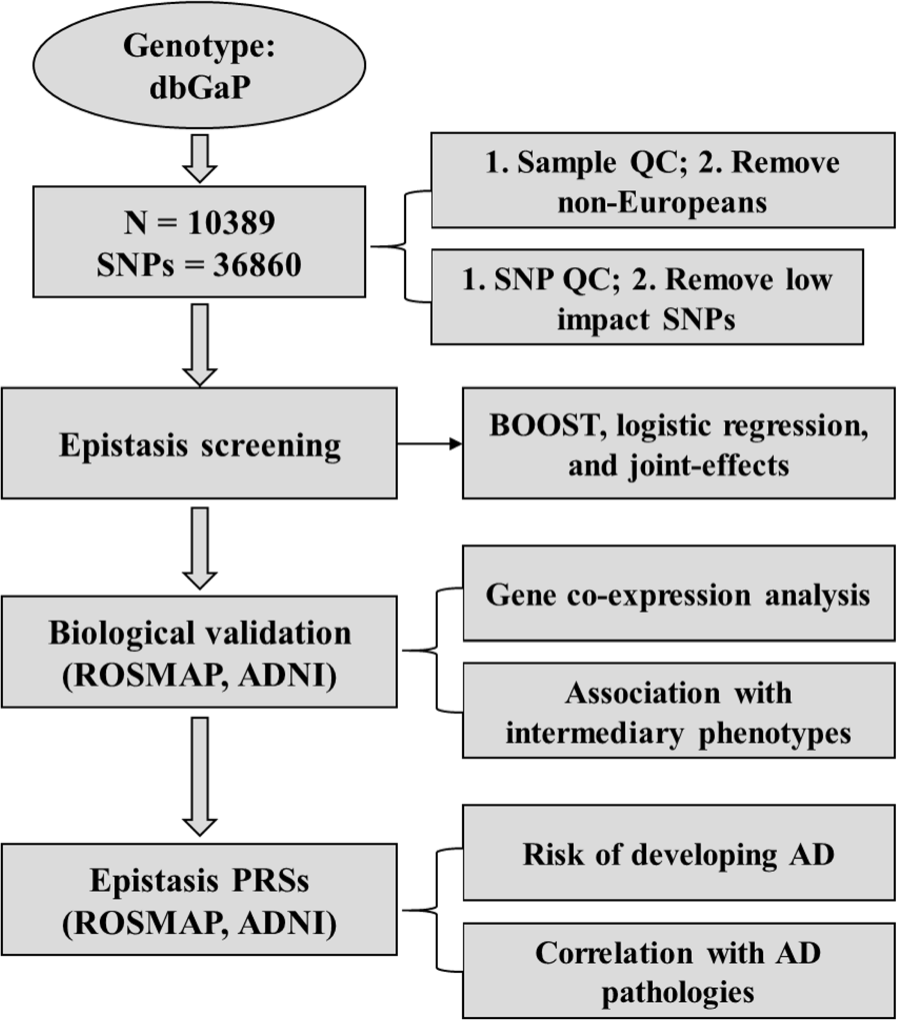
The workflow of our genetic interaction screening and validation procedures. AD, Alzheimer’s disease; BOOST, Boolean operation-based screening and testing; ADNI: the Alzheimer’s Disease Neuroimaging Initiative; dbGaP, the database of Genotypes and Phenotypes; PRS, polygenic risk score; ROSMAP: the Religious Orders Study and the Rush Memory and Aging Project; SNP, single nucleotide polymorphism

The polygenic architecture of AD enables the construction of predictive models based on genome-wide significant polymorphisms. A previous analysis of polygenic risk scores (PRSs) based on 22 GWAS-identified SNPs showed that PRSs were associated with the risk of AD and cerebrospinal fluid β-amyloid (1-42) (Aβ1-42) [17]. Another study showed that elevated PRSs were associated with worse memory and a smaller hippocampus at baseline, as well as greater longitudinal cognitive decline and clinical progression [18, 19]. In these analyses, PRSs displayed a significant but only moderate association with the trait of interest. Thus, PRSs alone seemed to be insufficient to capture the whole genetic landscape of AD. Through identifying genetic interactions, we attempted to evaluate the predictive capacities of epistasis risk scores as a complement to traditional PRSs. The whole workflow of our proposed analysis is displayed in Fig. 1.

## Methods

### Study subjects

Three independent datasets were used for genome-wide epistasis screening. National Institute on Aging (NIA)-LOAD (dbGaP accession: phs000168.v2.p2) study was to identify and recruit families with two or more siblings with the late-onset form of AD and a cohort of unrelated, non-demented controls similar in age and ethnic background, and to make the samples, the clinical and genotyping data and preliminary analyses available to qualified investigators worldwide [20]. Genetic Alzheimer’s Disease Associations (GenADA, dbGaP accession: phs000219.v1.p1) was a multi-site collaborative study to associate DNA sequence (allelic) variations in candidate genes with AD [21]. The NIA Alzheimer’s Disease Centers (ADCs, dbGaP accession: phs000372.v1.p1) cohort consisted of autopsy-confirmed and clinically confirmed AD cases, cognitively normal elders (CNEs) with complete neuropathology data who were older than 60 years of age at death and living CNEs who were documented to not exhibit mild cognitive impairment (MCI) and were between 60 and 100 years of age at assessment [22].

Two additional independent datasets were used for the construction and testing of PRSs. The Religious Orders Study and the Rush Memory and Aging Project (ROS/MAP) study were longitudinal clinical-pathologic cohort studies of AD [23]. The diagnosis of AD for each subject was performed by a neurologist who reviewed all available clinical data each year blinded to prior years and at the time of death for all years blinded to all pathologic data. The Alzheimer’s Disease Neuroimaging Initiative (ADNI, including phases 1, GO, and 2) was an international cooperative study conducted to investigate the pathology of AD and to develop treatments to slow or stop AD progression [24]. All subjects were administered clinical evaluations at the time of enrollment by expert physicians.

### Genotyping, imputation, and sample quality control

The NIA-LOAD samples were genotyped using the Illumina Human610-Quad BeadChip (Illumina, Inc., San Diego, CA, USA). The GenADA samples were genotyped using the Human Mapping 500 K Array Set (Affymetrix, Inc., Santa Clara, CA, USA). Samples from the ADCs were genotyped in two batches by using either the Human660W-Quad BeadChip or the HumanOmniExpress BeadChip (Illumina Inc., San Diego, CA, USA). There were 11,896 samples with both genotyping data and a clinical diagnosis of AD available in the three datasets.

Individuals in the ROS/MAP cohort were genotyped in two batches with a total sample size of 2090. The first batch was genotyped using the Affymetrix GeneChip 6.0 (Affymetrix, Inc., Santa Clara, CA, USA) at the Broad Institute’s Center for Genotyping or the Translational Genomics Research Institute. The other batch was genotyped using the Illumina HumanOmniExpress platform (Illumina, Inc., San Diego, CA, USA) at the Children’s Hospital of Philadelphia. A total of 1550 subjects from ADNI were genotyped with two platforms. A total of 757 individuals in ADNI1 were genotyped using the Illumina Human610-Quad BeadChip (Illumina, Inc., San Diego, CA, USA). A total of 793 ADNIGO/2 subjects were genotyped using the HumanOmniExpress BeadChip (Illumina Inc., San Diego, CA, USA).

All datasets were phased using Eagle (v2.4.1) [25] and imputed using Minimac3 [26]. Genotyping data were first aligned to the human GRCh37/hg19 assembly using UCSC’s liftOver tool [27]. Then, allele filtering and imputation were carried out as described in a previous study [28] with 1000 Genomes Phase3 integrated haplotypes as the reference panel [29]. Imputed variants with an imputation quality statistic (*R*^2^) below 0.3 were discarded.

Only individuals of European descent were selected for further analysis using GRAF-pop [30]. We excluded samples with a genotype missing rate > 0.2 or heterozygosity rate ± 3 standard deviations from the mean. We removed individuals with discordant sex information between the input dataset and those imputed from X chromosome inbreeding coefficients. Then, samples from NIA-LOAD, GenADA, and ADCs were merged into one large dataset (*N* = 10,389). Two batches from ROS/MAP were merged into one dataset (*N* = 2079). The ADNI1/GO/2 data were merged into one dataset, which will hereafter be referred to as the ADNI dataset (*N* = 1419). After sample quality control, 10,389 individuals were kept in the discovery dataset consisted of NIA-LOAD, GenADA, and ADCs (Table 1). For ROS/MAP, of 2090 individuals, 2079 were kept for further analysis (Table 2). For ADNI, of 1550 individuals, 1419 were kept for further analysis (Table 2).

**Table 1:** Characteristics of study participants (*N* = 10,389) after QC of genetic data

Cohort	AD			Non-AD		
	***N***	Age, years (SD)	Sex (F/M)	***N***	Age, years (SD)	Sex (F/M)
**NIA-LOAD**	1832	76.14 (7.10)	650 M, 1182 F	1986	76.38 (8.55)	790 M, 1196 F
**GenADA**	805	78.04 (8.60)	340 M, 465 F	779	73.41 (7.92)	278 M, 501 F
**ADCs**	3516	72.00 (9.27)	1655 M, 1861 F	1471	75.90 (9.56)	542 M, 929 F
**Total**	**6153**	**74.02 (8.92)**	**2645 M, 3508 F**	**4236**	**75.43 (8.96)**	**1610 M, 2626 F**

NIA-LOAD, National Institute of Aging-Late Onset Alzheimer’s Disease; GenADA, Genetic Alzheimer’s Disease Associations; ADCs, Alzheimer’s Disease Centers; AD, Alzheimer’s disease; F, female; M, male; SD, standard deviation

**Table 2:** Characteristics of study participants from ROS/MAP (N = 2079) and ADNI1/GO/2 (*N* = 1419) after QC of genetic data

**ROS/MAP**	**AD (*n*** **= 564)**	**Non-AD**^**a**^ **(*n*** **= 1515)**	**Diff (P)**^**b**^
Sex (F/M)	388 F, 176 M	1060 F, 455 M	0.63
Age at death, years (SD)	90.92 (5.88)	88.10 (6.73)	< 0.0001
*APOE* ε4 status (−/+)	− 378, + 186 (0.33)	− 1226, + 289 (0.19)	< 0.0001
**ADNI**	**AD (*n*** **= 555)**	**Non-AD (*n*** **= 864)**	**Diff (P)**^**b**^
Sex (F/M)	231 F, 324 M	372 F, 492 M	0.62
Age at AD, years (SD)	74.78 (8.11)	77.49 (7.33)	< 0.0001
*APOE* ε4 status (−/+)	−201, + 354 (0.64)	− 568, + 296 (0.34)	< 0.0001

ROS/MAP, The Religious Orders Study/the Rush Memory and Aging Project; ADNI, Alzheimer’s Disease Neuroimaging Initiative; AD, Alzheimer’s disease; Diff, the statistical difference between AD and non-AD; F, female; M, male; SD, standard deviation; Age at AD, age when AD developed for AD patients or age at last valid record for non-AD subjects; *APOE* ε4 status (−/+), presence of the ε4 allele

^a^Non-AD in ROS/MAP includes 713 individuals with missing AD status

^b^*P* values are calculated by Fisher’s exact tests (for sex and *APOE* ε4 status) or two-sample *t* tests (for age at death, age at AD)

### SNP selection and quality control

We selected SNPs that were more likely to be deleterious based on combined annotation-dependent depletion (CADD) scores [31]. After imputation, only SNPs that were located within 5 kb of any protein-coding gene with a CADD score ≥ 15 were retained for further analysis. Furthermore, calls with an uncertainty greater than 0.2 or import dosage certainty smaller than 0.8 were treated as missing using PLINK (v1.90b4.10) [32]. Then, SNPs with a missing rate > 0.1, minor allele frequency <  0.05, or Hardy-Weinberg equilibrium test value of *P* < 1 × 10^−6^ were removed. Ultimately, 36,860 SNPs passed the filtering and quality control processes.

### AD pathologies and neuroimaging

Intermediate phenotypes such as AD pathologies and neuroimaging data can help understand how the identified genetic interactions work. Subsets of samples from ROS/MAP and ADNI have AD pathologies and neuroimaging data available. In ROS/MAP, immunohistochemistry and automated image processing were used for the measurement of total amyloid and paired helical filament tau (PHF-tau). A modified Bielschowsky silver staining technique was used to measure neuritic plaques, diffuse plaques, and neurofibrillary tangles. Among 2079 subjects in ROS/MAP, at the time of these analyses, 1310 had available measurements of neurofibrillary tangles, neuritic plaques, and diffuse plaques; 1279 had available total PHF-tau measurements; and 1270 had available total amyloid measurements. In ADNI, cerebrospinal fluid (CSF) total tau (T-tau), phosphorylated tau (P-tau), and β-amyloid (1-42) (Aβ_1-42_) levels were measured via electrochemiluminescence immunoassays. Among 1419 individuals in the ADNI cohort, 1043 had available CSF T-tau, CSF P-tau, and CSF Aβ_1–42_ measurements. Molecular and structural neuroimaging data were also available for a subset of ADNI individuals. Structural magnetic resonance imaging was employed to generate estimates of the entorhinal cortex and hippocampal volume for 782 individuals. Fractional anisotropy (FA) for five bilateral fronto-temporal-occipital and interhemispheric white matter tracts (the sagittal stratum, hippocampal segment of the cingulum bundle, splenium of the corpus callosum, inferior fronto-occipital fasciculus, and superior longitudinal fasciculus) was estimated from diffusion-weighted images for 188 subjects.

### Association and interaction analysis

Association analysis was used to evaluate the main effects of selected SNPs. A linear mixed model [33] was employed to detect the association between SNPs and the AD status with sex, age, imputation batch, and the first three principal components as covariates. The discovery dataset consisted of data from three studies (NIA-LOAD, GenADA, and ADCs) in four batches. To avoid multicollinearity, only three dummy variables for four batches were used as covariates.

We employed three widely used models to test for significant genetic interactions in AD. The first method uses logistic regression models by including an additional interaction term [32]. The other two methods are faster in scanning for epistasis based on the inspection of 3 × 3 joint genotype count tables. Boolean operation-based testing and screening (BOOST) allows the use of fast logic (bitwise) operations to obtain contingency tables [34]. Joint-effect tests maintain correct type 1 error rates under the null hypothesis [35]. For logistic regression, BOOST, and joint-effects, SNP pairs with fewer than 3 observations in any 3 × 3 × 2 contingency table cell (cases and controls were considered separately) were removed from the analysis, resulting in 392,241,651 valid tests being performed. Multiple-testing correction for statistical tests across the three methods was conducted using false discovery rate (FDR). Post hoc analysis was performed using a genotypic test [36] adjusting for sex, age, imputation batches, and the first three principal components to ensure that these potential confounders had not caused any observed association. Then, linkage disequilibrium (LD) between each pair of genetic interaction was measured by *R*^2^. Genetic interactions with an LD > 0.2 were removed from further analysis.

The associations of candidate interactions with AD pathologies and neuroimaging phenotypes were analyzed using the same genotypic test [36] adjusted for sex, age, imputation batches, and the first three principal components.

### eQTL analysis and gene co-expression

Genetic interactions were further examined by eQTL and co-expression analysis. For eQTL analysis, data were obtained and analyzed using the Genotype-Tissue Expression (GTEx) web platform [37]. For co-expression analysis, SNPs were mapped to the nearest genes within a distance of 5 kb. RNA-Seq data were obtained from samples of the gray matter of the dorsolateral prefrontal cortex of 724 subjects from the ROS/MAP cohorts. These samples were quantified by using a Nanodrop spectrophotometer, and their quality was evaluated with an Agilent Bioanalyzer (Agilent Technologies, Inc., Santa Clara, CA, USA). A total of 582 RNA-Seq samples met the quality (Bioanalyzer RNA integrity (RIN) score > 5) and quantity (5 μg) thresholds. Then, the RNA-Seq data were processed via a parallelized automatic pipeline. The FPKMs were quantile normalized, and potential batch effects were removed by using the combat package in R. Pairwise correlations for gene co-expression were measured with the Pearson correlation coefficient.

### Definition of epistasis risk scores and combined risk scores

PRSs have been used to inform the disease risk of a patient for the early prevention of the disease [17, 38]. Given a set of SNPs, PRSs are derived by multiplying the number of risk alleles for each SNP by the natural logarithm of their respective odds ratios (ORs) and summing these products for each subject [39]. For the *j*^*th*^ individual, the PRS is defined by:

$$ {PRS}_j=\sum \limits_i\frac{G_{ij}\times {E}_i}{N_j} $$

where *E*_*i*_ is the effect size for the *i*^*th*^ SNP, *G*_*ij*_ is the number of effective alleles observed for the *i*^*th*^ SNP of *j*^*th*^ individual, and *N*_*j*_ is the number of SNPs included in the PRS for the *j*^*th*^ individual.

For risk scores defined by epistasis, there is no readily available definition from previous publications. Inspired by an epistasis analysis framework called multifactor dimensionality reduction (MDR) [40, 41], we define the epistasis risk score (ERS) for the *j*^*th*^ individual as:

$$ {ERS}_j=\sum \limits_i\frac{\sum \limits_{k=1}^9{C}_{ik}{E}_{ik}}{N_j} $$

where *C*_*ik*_ equals to 1 if the *j*^*th*^ individual was assigned to the *k*^*th*^ cell of the 3 × 3 genotype contingency table for the *i*^*th*^ interaction. Otherwise, *C*_*ik*_ equals to 0. *E*_*ik*_ is the effect size (natural logarithm of OR) of the *k*^*th*^ cell of 3 × 3 genotype contingency table for the *i*^*th*^ interaction. *N*_*j*_ is the number of interactions included in the ERS for the *j*^*th*^ individual.

The combined risk score (CRS) of SNPs and SNP-SNP interactions for the *j*^*th*^ individual is defined as:

$$ {CRS}_j=w\times {PRS}_j+\left(1-w\right)\times {ERS}_j $$

where *w* is a weighting factor for PRS and ERS. To avoid using an arbitrary *w*, for individuals from ADNI, we selected w that maximized the AUC of CRSs in ROS/MAP. Likewise, we selected w that maximized the AUC of CRSs in ADNI for individuals from ROS/MAP.

### Evaluation of AD risk using genetic interactions

We constructed several sets of risk scores to evaluate the contribution of epistasis to disease risk. For PRSs, *APOE* (rs7412 and rs429358) and 20 common SNPs identified by a previous GWAS [5] were included in the analysis. The effect size of each SNP was also obtained from the same study [5]. For ERSs, the effect size (natural logarithm of odds ratio) of each cell in the 3 × 3 genotype contingency table for each genetic interaction was obtained on the discovery data (*N* = 10,389). Based on *P* value cutoff of 1 × 10^−7^, 1 × 10^−6^, or 1 × 10^−5^, three sets of ERSs were constructed. Moreover, the predictive power of each nominal significant genetic interaction (*P* < 1 × 10^−5^) was evaluated via a permutation test. For each genetic interaction, an MDR model [41] was trained using the discovery data. Based on the MDR model, predictions of AD status were given for each genetic interaction on the two testing datasets, i.e., ROS/MAP and ADNI. The predictions for each interaction were permuted for 10,000 times in each testing dataset to generate a null distribution of random predictions. Based on the null distribution, we selected 77 interactions that showed non-random effects (*P* <  0.05) in both ROS/MAP and ADNI.

To evaluate whether the genetic risk scores were associated with age at onset of AD, we divided samples into 4 quantiles based on the corresponding genetic risk scores. The age at onset of AD in each quantile was analyzed with the Kaplan-Meier method, where patients were censored at the last record entry. The differences in the age at onset of AD in 4 quantiles were compared statistically using the log-rank test. Furthermore, the receiver operating characteristic (ROC) curves were generated by plotting the true-positive rate against the false-positive rate. Then, the area under the ROC curve (AUC) was calculated for each ROC curve to quantify the prediction accuracy of each type of genetic risk score.

## Results

### Genome-wide epistasis screening

**Fig. 2:**
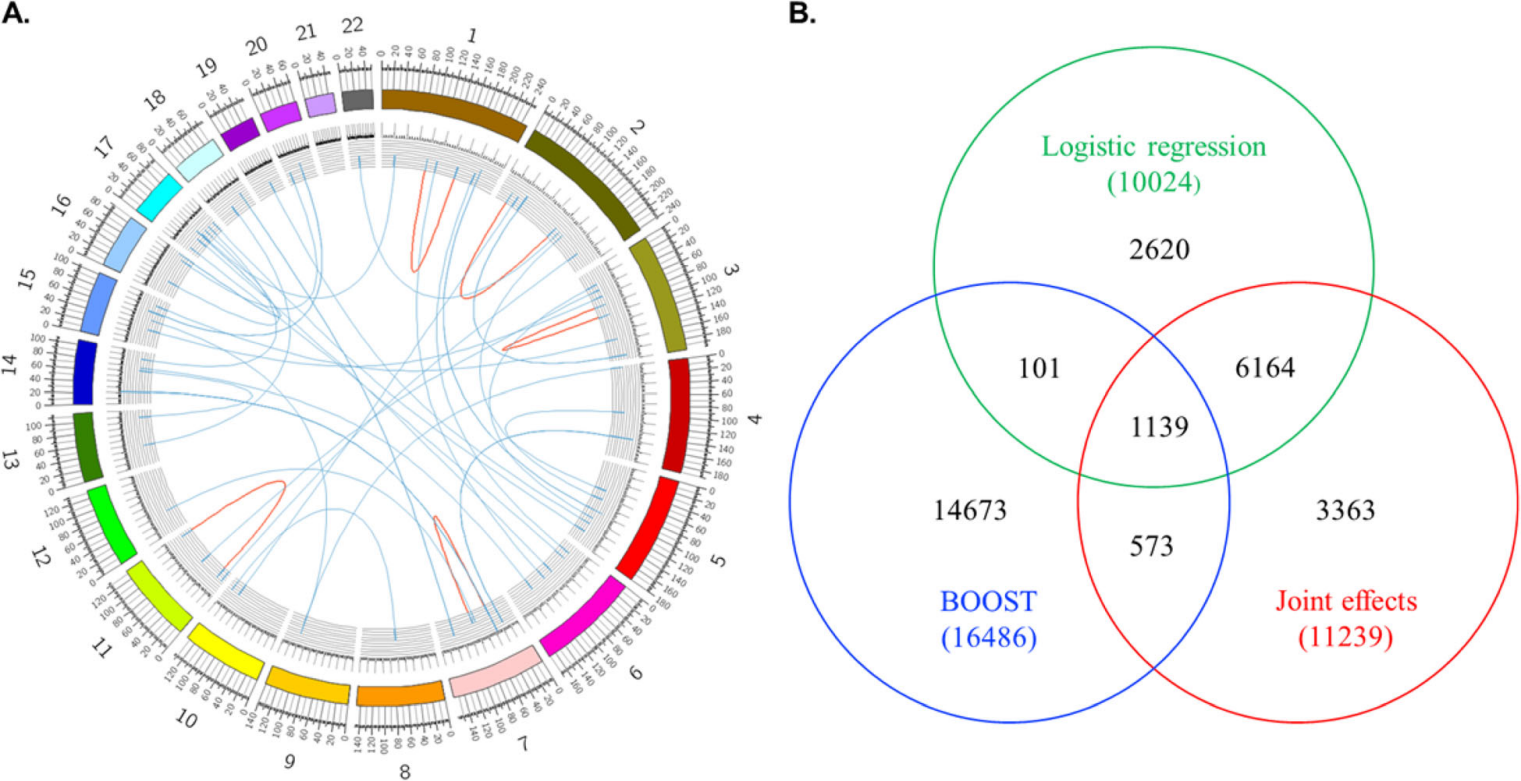
Genetic interactions identified by the three adopted methods. **a** Interactions with *P* value smaller than 1 × 10^−8^ are shown. Interactions within the same chromosome are marked in red. The histogram shows the interaction density. **b** There are 1139 common genetic interactions identified by all three methods (*P* < 1 × 10 ^−5^). Genetic interactions identified by BOOST are often different from the other two methods

First, we analyzed the main effect for each SNP using traditional genome-wide association analysis (GWAS). No genomic inflation was observed on the Q-Q plot (Figure S1). One genome-wide significant signal appeared nearby *APOE* on chromosome 19 (Figure S1). Epistasis screening for the clinical diagnosis of AD was carried out using three different methods. Under the BOOST model, a total of 16,486 SNP-SNP interactions (Table S1) were retained under a nominal *P* threshold of 1 × 10^−5^ For logistic regression and joint-effect tests, 11,239 and 10,024 SNP-SNP interactions (Tables S2 and S3) were retained under a nominal *P* threshold of 1 × 10^−5^, respectively. Overall, there were 28,633 SNP-SNP interactions identified by three methods (Fig. 2a). The SNP-SNP interactions obtained via different methods showed distinct results, with a higher overlap between logistic regression and joint effect tests (Fig. 2b).

Two significant SNP-SNP interactions were identified (*P*_*FDR*_ <  0.05) by logistic regression. The first interaction was between rs6952399 (chr7: 45210711, *RAMP3*: intron variant) and rs6974494 (chr7: 83743961, *SEMA3A*: intron variant). The other was between rs17856580 (chr16: 27246617, *NSMCE1*: missense variant) and rs1048159 (chr17: 54912339, *DGKE*: synonymous variant, *C17orf67*: upstream variant). Under a less conservative *P*_*FDR*_ of 0.1, the interaction between rs2164808 (chr2: 25377176, *EFR3B*: stop gained, *RP11-509E16.1*: upstream variant) and rs354709 (chr2:143886953, *ARHGAP15*: intron variant) was the only genetic interaction identified by BOOST. Five genetic interactions (*P*_*FDR*_ <  0.1) were identified by joint effect analysis, including rs6952399-rs6974494 which was also identified by logistic regression. No main effects were observed for these genetic interactions with an *P*_*FDR*_ <  0.1, except a nominal significant signal (*P* = 0.017) for rs2301600 in rs4574537-rs2301600. Interactions (*P*_*FDR*_ <  0.1) identified by three methods were displayed in Table 3.

**Table 3:** Significant genetic interactions with a false discovery rate (FDR) < 0.1

SNP1	Chr_1	Position_1^**a**^	SNP2	Chr_2	Position_2^**a**^	***P***	FDR	Gene_1	Gene_2
**Genetic interaction identified by BOOST**									
rs354709	2	143886953	rs2164808	2	25377176	1.47E−10	0.058	*ARHGAP15*	*EFR3B; RP11-509E16.1*
**Genetic interactions identified by logistic regression**									
rs6952399	7	45210711	rs6974494	7	83743961	1.88E−10	0.048	*RAMP3*	*SEMA3A*
rs17856580	16	27246617	rs1048159	17	54912339	2.43E−10	0.048	*NSMCE1*	*DGKE; C17orf67*
**Genetic interactions identified by joint effect**									
rs6952399	7	45210711	rs6974494	7	83743961	5.08E−10	0.076	*RAMP3*	*SEMA3A*
rs217362	7	44618599	rs8004063	14	23732479	7.61E−10	0.076	*DDX56; TMED4*	*C14orf164; RNU6-1046P*
rs4574537	5	137419728	rs2301600	19	35786868	7.70E−10	0.076	*WNT8A*	*MAG*
rs2075302	2	163076146	rs374479	5	80998915	8.03E−10	0.076	*FAP*	*SSBP2*
rs8580	7	44620836	rs8004063	14	23732479	9.70E−10	0.076	*TMED4*	*C14orf164; RNU6-1046P*

Chr_1, chromosome of SNP1; Gene_1, genes within 5 kb of SNP1; Chr_2, chromosome of SNP2; Gene_2, genes within 5 kb of SNP2;

^a^SNP positions were in build 37, assembly hg19

### Transcription analysis of candidate interactions

**Fig. 3:**
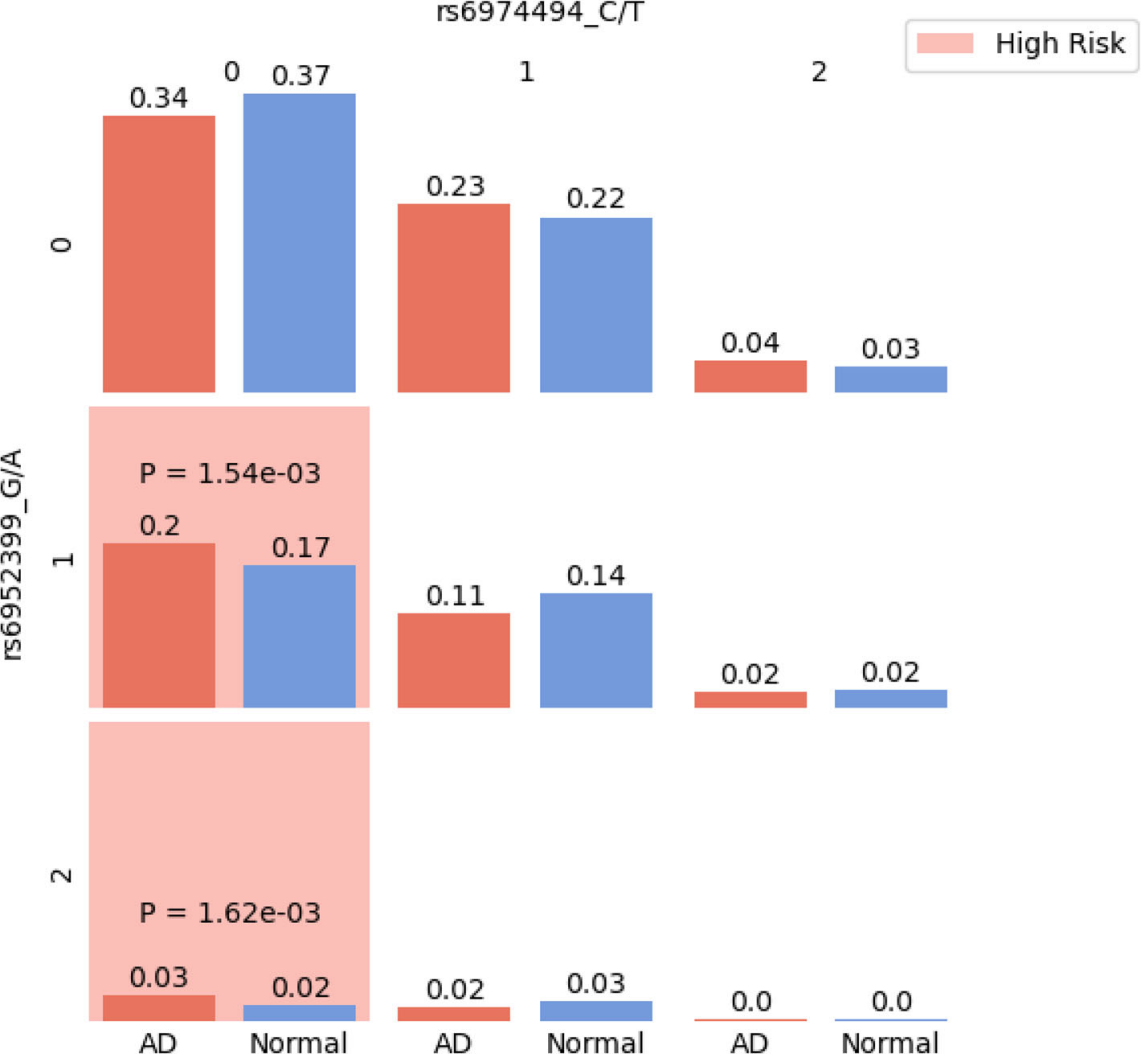
Visualization of rs6952399-rs6974494 interaction. The ratio of case and control in each cell is shown. Cells with significantly higher cases than controls by fisher’s exact test are marked red. The counted allele for rs6952399 is G. The counted allele for rs6974494 is C

The expression levels of genes that interact with each other are likely to be positively or negatively correlated [42]. Combining the interaction pattern with co-expression and eQTL analysis, we can gain biological insight beyond the statistical significance. Visualization of the genetic interaction showed that rs6952399^G^-rs6974494^TT^ carriers displayed a higher risk of developing AD (Fig. 3). Based on eQTL analysis, rs6952399^G^ carrier showed a higher expression of *RAMP3* (*P* = 2.4 × 10^−6^), and rs6974494^T^ carrier showed a lower expression of *SEMA3A* (*P* = 1.6 × 10^−5^). Therefore, it is likely that the upregulation of *RAMP3* expression combined with the downregulation of *SEMA3A* confers a higher risk of AD (Fig. 3). This assumption is further supported by the fact that *RAMP3* and *SEMA3A* showed negative co-expression in the brain (*R* = − 0.29). Moreover, *RAMP3* and *SEMA3A* demonstrated higher negative correlation in AD patients (*R* = − 0.33) compared to cognitive normal controls (*R* = − 0.24).

The seven SNP-SNP interactions identified by the three models correspond to nine gene-gene interactions, five of which showed significant co-expression. Besides the co-expression between *RAMP3* and *SEMA3A,* a negative correlation has been observed between *NSMCE1* and *DGKE*/*C17orf67* and between *ARHGAP15* and *EFR3B*/*RP11-509E16.1* as well (Table S4). Statistical epistasis often lacks biological interpretation. Here, we provide the visualization of genetic interactions (Fig. 3, Figure S2) together with the results of co-expression and eQTL analysis (Table S4) to facilitate biological interpretation of statistical significance.

### Candidate interactions and AD neuropathology

To investigate the biological mechanism of the identified genetic interactions, we tested the associations of these interactions (*P*_*FDR*_ < 0.1) with intermediate AD phenotypes including Aβ and tau protein levels, brain atrophy, and white matter injury. None of the candidate genetic interactions displayed a significant association with the entorhinal cortex or hippocampal volume. One interaction, between rs8580 (chr7: 44620836, *TMED4*: synonymous variant) and rs8004063 (chr14: 23732479, *C14orf164*: intron variant), demonstrated significant association with neurite plaques in the entorhinal cortex (*P* = 0.019). Due to linkage disequilibrium between rs217362 and rs8580, the rs217362-rs8004063 interaction also manifested a significant association with neurite plaques in the entorhinal cortex (*P* = 0.023). TMED4 belongs to p24 family proteins, which are mainly involved in vesicular protein trafficking and are likely to promote neuritic plaque formation in AD [43]. *C14orf164* is an important paralog of *RNF212* which can encode an E3 enzyme in the ubiquitin proteasome system whose dysfunction could lead to Aβ accumulation [44, 45]. Their molecular function supports that the interaction may act through the trafficking and processing of APP in AD pathogenesis.

White matter (WM) fractional anisotropy (FA) is thought to be related to WM integrity, and a decline in FA is often used as an index of decreasing WM health. The interaction between rs2164808 (chr2: 25377176, *EFR3B*: stop gained, *RP11-509E16.1*: upstream variant) and rs354709 (chr2: 143886953, *ARHGAP15*: intron variant) showed significant associations (left *P* = 0.046, right *P* = 0.023) with FA estimates in the splenium of the corpus callosum. EFR3B and phosphatidylinositol 4-kinase alpha (PI4KA) forms a protein complex that plays an important role in the myelination process via actin dynamics [46]. Interestingly, ARHGAP15, which is a Rac-specific negative regulator, is also heavily involved in actin cytoskeleton dynamics [47], suggesting that its interaction with EFR3B affects WM health in AD patients through regulating myelination in axons.

### Epistasis risk scores in AD

The polygenic basis of LOAD can be harnessed to identify individuals at risk for cognitive decline. Previously, PRSs were inferred from the cumulated effects of each disease-associated SNP. We investigated whether ERSs that were inferred from the summed effects of each disease-associated SNP-SNP interactions could serve as an indicator of disease risk. After removing redundant genetic interactions due to LD, 19,264 of 28,633 genetic interactions with *P* < 1 × 10^−5^ were kept for ERS analysis. ERSs were constructed based on three different *P* value thresholds: *P* < 1 × 10^−7^ (298 interactions), *P* < 1 × 10^−6^ (2478 interactions), and *P* < 1 × 10^−5^ (19,264 interactions).

**Fig. 4:**
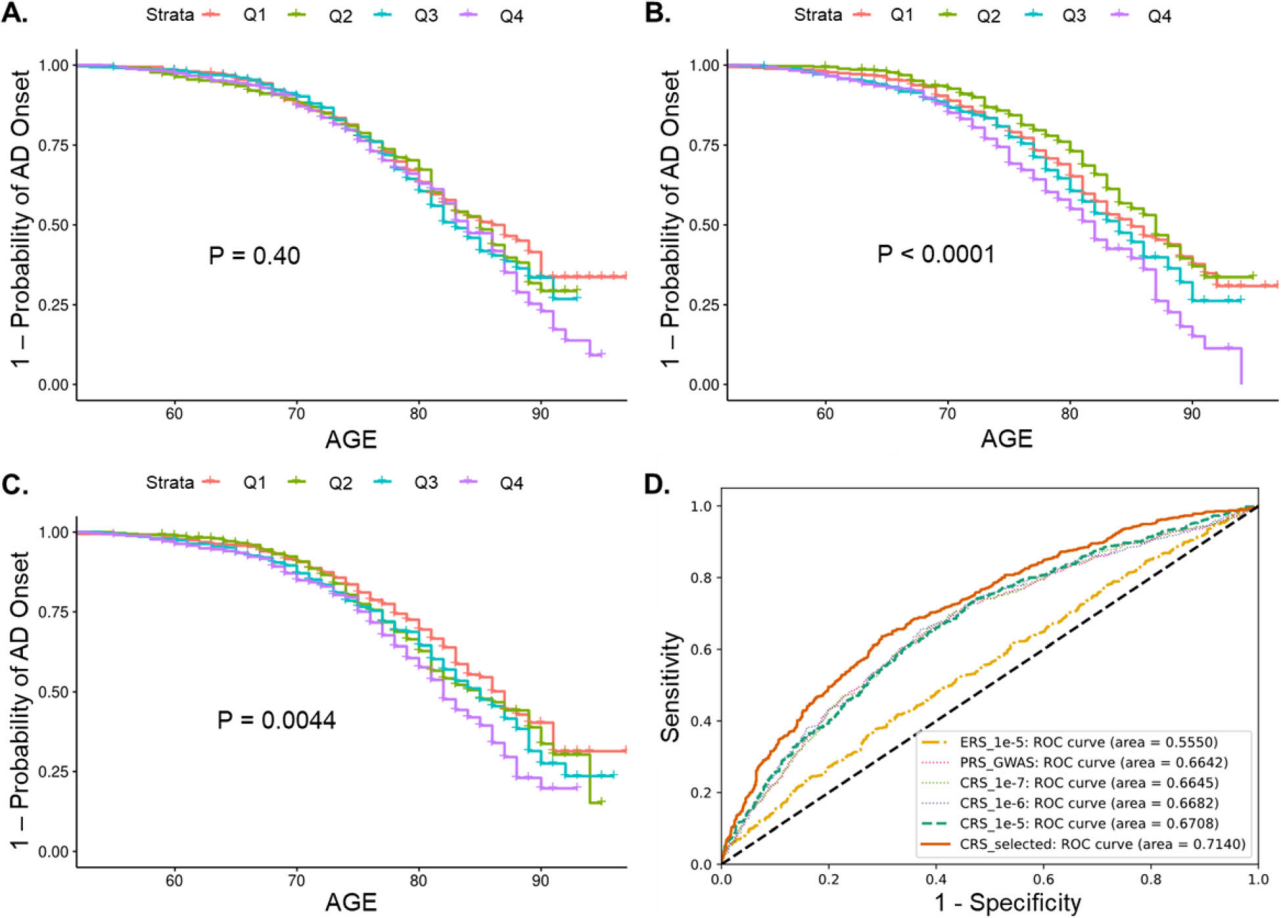
Performance of epistasis risk scores (ERSs), polygenic risk scores (PRSs), and combined risk scores (CRSs) in AD risk prediction using samples from ADNI. Samples were divided into four quantiles (Q1 to Q4: from the lowest risk to the highest risk) based on their ERSs. The probability of developing AD was analyzed by the Kaplan-Meier method, where the *P* value was obtained by the log-rank test. ERSs were obtained via interactions with **a** *P* < 1 × 10^−7^ (298 interactions), **b** *P* < 1 × 10^−6^ (2478 interaction), or **c** *P* < 1 × 10^−5^ (19,264 interactions). **d** Comparison of AUCs of ERSs, PRSs, and CRSs in identifying AD patients. ERS_1e-5: ERSs constructed by genetic interactions with *P* value smaller than 1 × 10^−5^; PRS_GWAS: PRSs constructed by *APOE* (rs7412 and rs429358) and 20 SNPs identified by previous GWAS; CRS_1e-7, CRS_1e-6, CRS_1e-5: combined risk score of SNPs and SNP-SNP interactions with *P* value smaller than 1 × 10^−7^, 1 × 10^−6^, or 1 × 10^−5^; CRS_selected: similar to CRS_1e-5, except that only 77 genetic interactions showing non-random effects in ROS/MAP and ADNI were included

We evaluated if individuals with higher ERSs had a higher risk of AD, therefore, had onset of AD at an earlier age. It was shown that ERSs constructed from genetic interactions with *P* < 1 × 10^−7^ could not identify high-risk individuals from low-risk individuals in either ADNI or ROS/MAP (Fig. 4a; Figure S3). This may arise from the fact that SNPs or SNP-SNP interactions identified from association analysis often had a very small effect size. Adding more genetic interactions (e.g., 2478 interactions or 19,264 interactions) gradually increased the power of ERSs to stratify high risk individuals from low risk individuals (log-rank test *P* < 0.0001, Fig. 4b; log-rank test *P* = 0.0044, Fig. 4c; Figure S3). Thus, we demonstrated that ERS could serve as an indicator of the genetic risk of AD (Fig. 4; Figure S3). The same conclusion still held true when samples from ROS/MAP were used (Figure S3). Furthermore, we evaluated if the predictive power of ERSs was due to SNPs that have a main effect in AD. We found that ERSs still demonstrated the power to stratify high-risk individuals from low-risk individuals, after removing interactions that contained any main effect SNP (*P* < 0.05) (Figure S4).

### Combined risk scores of SNPs and SNP-SNP interactions

We evaluated if combined risk scores (CRS) of SNPs (defining PRS) and SNP-SNP interactions (defining ERS) could be a better indicator of AD risk. PRSs and ERSs showed an AUC of 0.66 and 0.56, respectively (Fig. 4d). When ERSs consisting of genetic interactions with *P* < 1 × 10^−7^, 1 × 10^−6^, or 1 × 10^−5^ were combined with PRSs for the construction of CRSs, the AUC of CRSs showed a non-significant but steady increase up to 0.67 (Fig. 4d). The similar non-significant but steady increase of AUC for CRSs obtained using different *P* value thresholds were also detected using individuals from ROS/MAP (Figure S3).

**Fig. 5:**
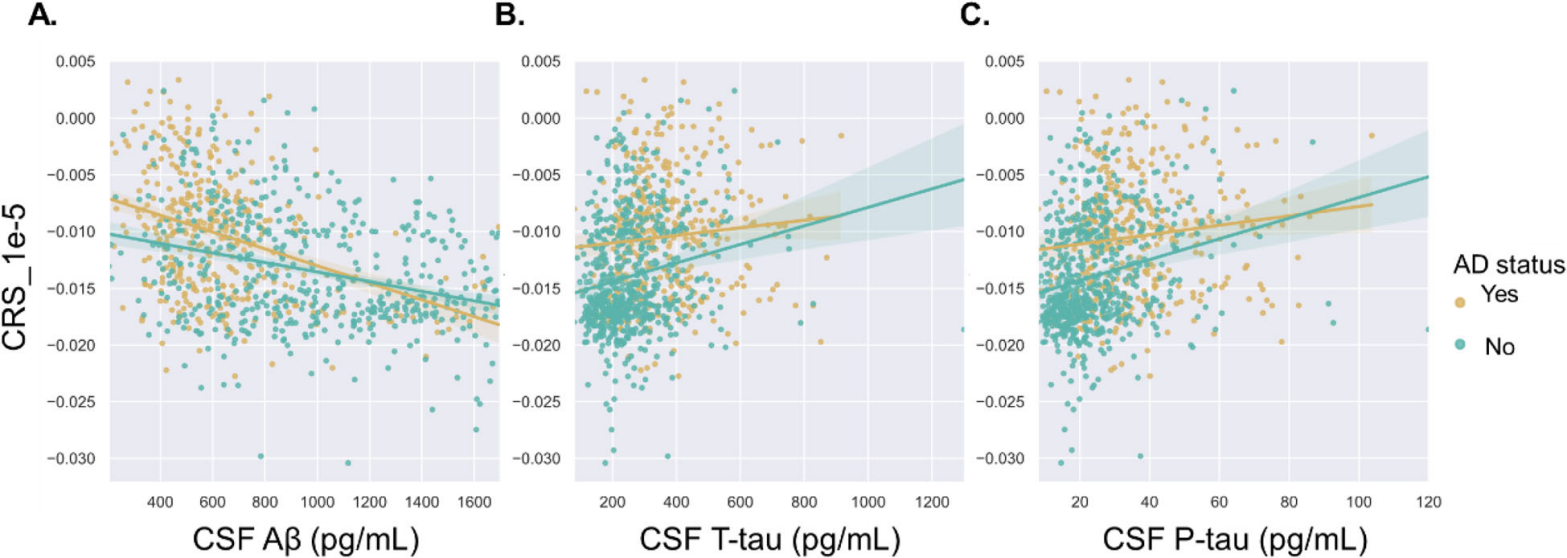
Associations between CRSs_1e-5 (combined risk scores constructed by genetic interactions with *P* < 1 × 10^−5^) and Alzheimer’s disease pathologies. **a** CRSs were negatively correlated with CSF Aβ_1–42_ (AD (*n* = 388): *R* = −0.41, *P* = 8.4 × 10^−17^; non-AD (*n* = 655): *R* = − 0.35, *P* = 7.1 × 10^−20^). **b** CRSs showed a positive correlation with CSF total tau (AD (*n* = 388): *R* = 0.088, *P* = 0.082; non-AD (*n* = 655): *R* = 0.19, *P* = 5.8 × 10^−7^). **c** CRSs showed a positive correlation with phosphorylated tau (AD (n = 388): *R* = 0.12, *P* = 0.018; non-AD (n = 655): *R* = 0.23, *P* = 2.3 × 10^−9^)

We evaluated the correlation between CRSs derived from genetic interactions with *P* < 1 × 10^−5^ and CSF markers of AD. It was found that CRSs displayed a strong negative correlation with CSF β-amyloid (1–42) (*R* = − 0.40, *P* = 1.8 × 10^−35^) and a strong positive correlation with CSF total tau (*R* = 0.24, *P* = 2.2 × 10^−15^) and CSF phosphorylated tau (*R* = 0.27, *P* = 2.1 × 10^−19^) (Fig. 5). Interestingly, CRSs showed much stronger correlation with CSF total tau (AD: *R* = 0.088, *P* = 0.082; non-AD: *R* = 0.19, *P* = 5.8 × 10^−7^) and CSF phosphorylated tau (AD: *R* = 0.12, *P* = 0.018; non-AD: *R* = 0.23, *P* = 2.3 × 10^−9^) in cognitive normal controls than in AD patients (Fig. 5b, c). However, a higher correlation between CRSs and CSF β-amyloid (1–42) (AD: *R* = − 0.41, *P* = 8.4 × 10^−17^; non-AD: *R* = − 0.35, *P* = 7.1 × 10^−20^) was observed in AD patients (Fig. 5a). This also held true when PRSs were used in the correlation analysis (Figure S5). These results strongly suggest that tau may act in the earlier stage of AD as high-risk individuals showed faster accumulation of CSF tau when they still displayed normal cognitive status.

It should be noted that CRSs did not display a higher correlation with CSF markers compared with PRSs (Fig. 5; Figure S5). A previous study has discovered that APOE contributed mostly to amyloid accumulation and other SNPs only affected the risk of further conversion to AD [48]. The present analysis also reveals that genetic interactions affected the risk of AD rather than pathological markers.

Furthermore, we selected interactions that demonstrated the power to predict the risk of AD by itself alone (Methods). From 19,264 interactions with *P* < 1 × 10^−5^, 77 interactions that displayed predictive effects in both ROS/MAP and ADNI were selected. Combined risk scores of SNPs and these 77 interactions performed much better in predicting the clinical status of AD (ADNI: AUC = 0.71, Fig. 4d; ROSMAP: AUC = 0.69, Figure S3). However, because the testing datasets (ADNI and ROS/MAP) were used for selection, the prediction accuracy of this subset of interactions should be interpreted with caution. Their effects on risk prediction need to be evaluated when a new dataset is available. Overall, they represented a subset of SNP-SNP interactions, each of which alone demonstrated predictive capacity across our discovery and testing data. The full list of the selected interactions is shown in Table S5.

## Discussion

To help explain the missing heritability in AD, we performed a genome-wide interaction analysis of AD. There were seven candidate genetic interactions (*P*_*FDR*_ < 0.1) identified using the three most popularly adopted methods. Previous reports supported possible functional convergence between pairs of genes identified by our analysis, such as *RAMP3*-*SEMA3A* and *NSMCE1-DGKE*. It was reported that RAMP3, a component of amylin receptor-3, could induce cell death via neurotoxic actions of Aβ [49]. Semaphoring 3A (Sema3A) could bind to nonamyloidogenic sAPPα which would prevent the collapse of axonal growth cones induced by Sema3A [50]. Consequently, biological interaction between RAMP3 and Sema3A is likely to be involved in the neurodegeneration process of AD. For NSMCE1 and DGKε, there may exist direct physical interaction between them, as two independent studies have uncovered the exact same ubiquitination site at lysine 357 in human DGKε [51, 52]. NSMCE1 is a RING-type zinc finger-containing E3 ubiquitin ligase that assembles with melanoma antigen protein to catalyze the direct transfer of ubiquitin from E2 ubiquitin-conjugating enzyme to a specific substrate. DGKε is a membrane-bound diacylglycerol kinase that converts diacylglycerol into phosphatidic acid.

Moreover, we visualized the 3 × 3 contingency table of each interaction (Fig. 3; Figure S2). We attempted to combine the observed interaction pattern with the gene expression pattern (i.e., co-expression and eQTL) to infer the mechanism of action of each interaction. In this way, we found that the higher expression of *RAMP3* combined with the lower expression of *SEMA3A* conferred a higher risk of AD. Then, we related candidate interactions with intermediate phenotypes in AD such as Aβ and tau levels, brain atrophy, and FA estimates to help understand the biological consequences of the statistical significance. The association between *TMED4-C14orf164* and neurite plaques in the entorhinal cortex indicates that ubiquitination may play an important role in Aβ accumulation, as *C14orf164* is an important paralog of *RNF212* which can encode an E3 enzyme in the ubiquitin proteasome.

Epistasis has never been used in the construction of genetic risk scores. Here, we demonstrated that ERSs were able to discriminate high-risk individuals that were more likely to develop AD (Fig. 4; Figure S3). Then, combined risk scores of SNP and SNP-SNP interactions showed slightly but steadily increased AUC in predicting the clinical status of AD (Fig. 4d). Additionally, we selected a subset of 77 genetic interactions that showed non-random effects in both ROS/MAP and ADNI. It was shown that combined risk scores including the 77 interactions performed better in predicting the clinical status of AD than using all the genetic interactions with *P* < 1 × 10^−5^ (Fig. 4d; Figure S3). This indicated the possibility of combining PRSs and ERSs as potential biomarkers of AD. However, further evaluation of the selected interactions on new datasets is needed. Altogether, we demonstrated that ERS is a promising complement to traditional PRS in practical application.

## Limitations

To reduce the search space, we only analyzed SNPs with a CADD score ≥ 15 that were more likely to be causative. However, it is still possible for two non-deleterious SNPs to be disease-causing variants when there is a genetic interaction between them. In that case, faster tests are needed to test the interactions between millions of SNPs. Moreover, tests for interactions are complicated by the fact that samples are stratified by the 3 × 3 genotype contingency table. Therefore, cells with very small sample sizes are likely to induce false positives in the test results. We try to avoid this issue by removing pairs that show few samples in any cells of the 3 × 3 genotype contingency table. Thus, we are likely to have removed some rare allele pairs that might interact with each other. Overall, the predictive power of our model was based on a selected subset of deleterious common variants; further improvement may be expected when non-deleterious or rare variants could be incorporated.

Moreover, we simply combined PRSs and ERSs by a weighting factor. There may be a more complicated relationship between the additive effects of single SNPs and genetic interactions. The development of a better integrated model that can account for both main effects and epistasis would further increase the prediction accuracy of the genetic risk score.

## Conclusions

In conclusion, through a genome-wide epistasis analysis, we identified a number of genetic interactions that are often co-expressed and can partly explain the “missing heritability” in AD. Subsequent analysis revealed possible links between these genetic interactions and pathological endophenotypes. Furthermore, it was demonstrated that ERSs can identify high-risk individuals showing earlier onset of AD. Combined risk scores of SNPs and SNP-SNP interactions showed slightly but steadily increased AUC in predicting the clinical status of AD.

## Supplementary Information

**Additional file 1: Figure S1.** GWAS analysis of selected SNPs (N = 10389, SNPs = 36860). **Figure S2.** Visualization of SNP-SNP interactions. **Figure S3.** Performance of epistasis risk scores (ERSs), polygenic risk scores (PRSs) and combined risk scores (CRSs) in AD risk prediction using samples from ROS/MAP. **Figure S4.** Epistasis risk scores (ERSs) analysis after removing genetic interactions that showed main effects (P < 0.05) using samples from ADNI and ROS/MAP. **Figure S5.** Associations between PRSs (polygenic risk scores constructed by APOE, i.e., rs7412 and rs429358 and 20 SNPs identified by previous GWAS) and Alzheimer’s disease pathologies.

**Additional file 2: Table S1.** Nominal significant SNP-SNP interactions (BOOST). **Table S2.** Nominal significant SNP-SNP interactions (Logistic regression). **Table S3.** Nominal significant SNP-SNP interactions (Joint effect). **Table S4.** Co-expression and eQTL analysis of significant genetic interactions (FDR < 0.1). **Table S5.** Selected genetic interactions that showed non-random prediction in both ADNI and ROSMAP.

## Data Availability

Genotypes and phenotypes of samples from NIA-LOAD, GenADA, and ADCs can be accessed from dbGaP database (https://dbgap.ncbi.nlm.nih.gov) under the accession number of phs000168.v2.p2, phs000219.v1.p1, and phs000372.v1.p1, respectively. Genotypes, gene expression profiles, and post-mortem pathological measurements of AD patients from ROS/MAP can be obtained through RADC (https://www.radc.rush.edu). Genotypes, CSF pathological measurements, and neuroimages of samples from ADNI can be applied from http://adni.loni.usc.edu.

## Abbreviations

Aβ: β-amyloid
AD: Alzheimer disease
ADC: National Institute on Aging Alzheimer’s Disease Center
ADNI: Alzheimer’s Disease Neuroimaging Initiative
AUC: Area under the receiver operating characteristic curve
BOOST: Boolean operation-based testing and screening
CADD: Combined annotation-dependent depletion
CRS: Combined risk score
CSF: Cerebrospinal fluid
eQTL: Expression quantitative trait loci
ERS: Epistasis risk score
GenADA: Genetic Alzheimer’s Disease Associations
GWAS: Genome-wide association studies
LD: Linkage disequilibrium
LOAD: Late-onset Alzheimer disease
NIA-LOAD: National Institute on Aging-Late-Onset-Alzheimer’s-Disease Study
PRS: Polygenic risk score
ROS/MAP: The Religious Orders Study and the Rush Memory and Aging Project
SNP: Single nucleotide polymorphisms
