# Supplementary material for: Genome-wide epistasis analysis for Alzheimer’s disease and implications for genetic risk prediction
Source: Alzheimers Res Ther. 2021 Mar 4;13:55. doi: 10.1186/s13195-021-00794-8 (PMC7934265; doi:10.1186/s13195-021-00794-8)
Supplement: Supplementary file 1 — Additional file 1: Figure S1. GWAS analysis of selected SNPs (N = 10389, SNPs = 36860). Figure S2. Visualization of SNP-SNP interactions. Figure S3. Performance of epistasis risk scores (ERSs), polygenic risk scores (PRSs) and combined risk scores (CRSs) in AD risk prediction using samples from ROS/MAP. Figure S4. Epistasis risk scores (ERSs) analysis after removing genetic interactions that showed main effects (P < 0.05) using samples from ADNI and ROS/MAP. Figure S5. Associations between PRSs (polygenic risk scores constructed by APOE, i.e., rs7412 and rs429358 and 20 SNPs identified by previous GWAS) and Alzheimer’s disease pathologies. [file 13195_2021_794_MOESM1_ESM.docx]

**Supplementary Figures**

**
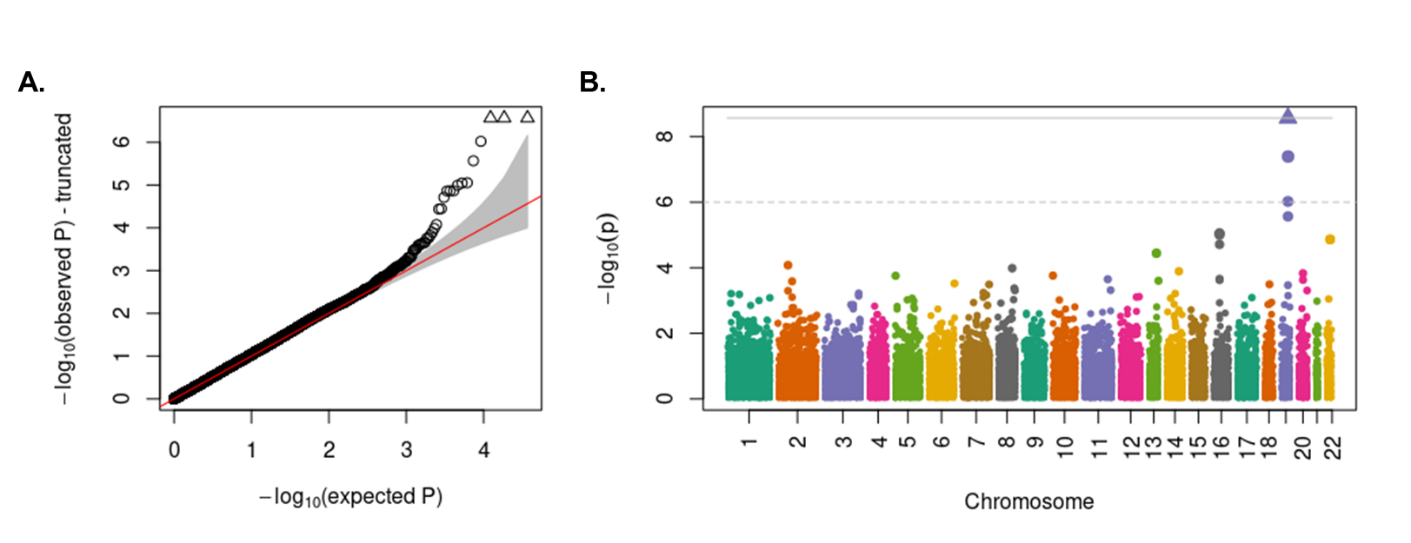
**

**Figure S1. GWAS analysis of selected SNPs (N = 10389, SNPs = 36860). (A)** Q-Q plot for GWAS *P* values. No genomic inflation is observed. **(B)** Manhattan plot shows one genome-wide significant region on chromosome 19 near *APOE*.


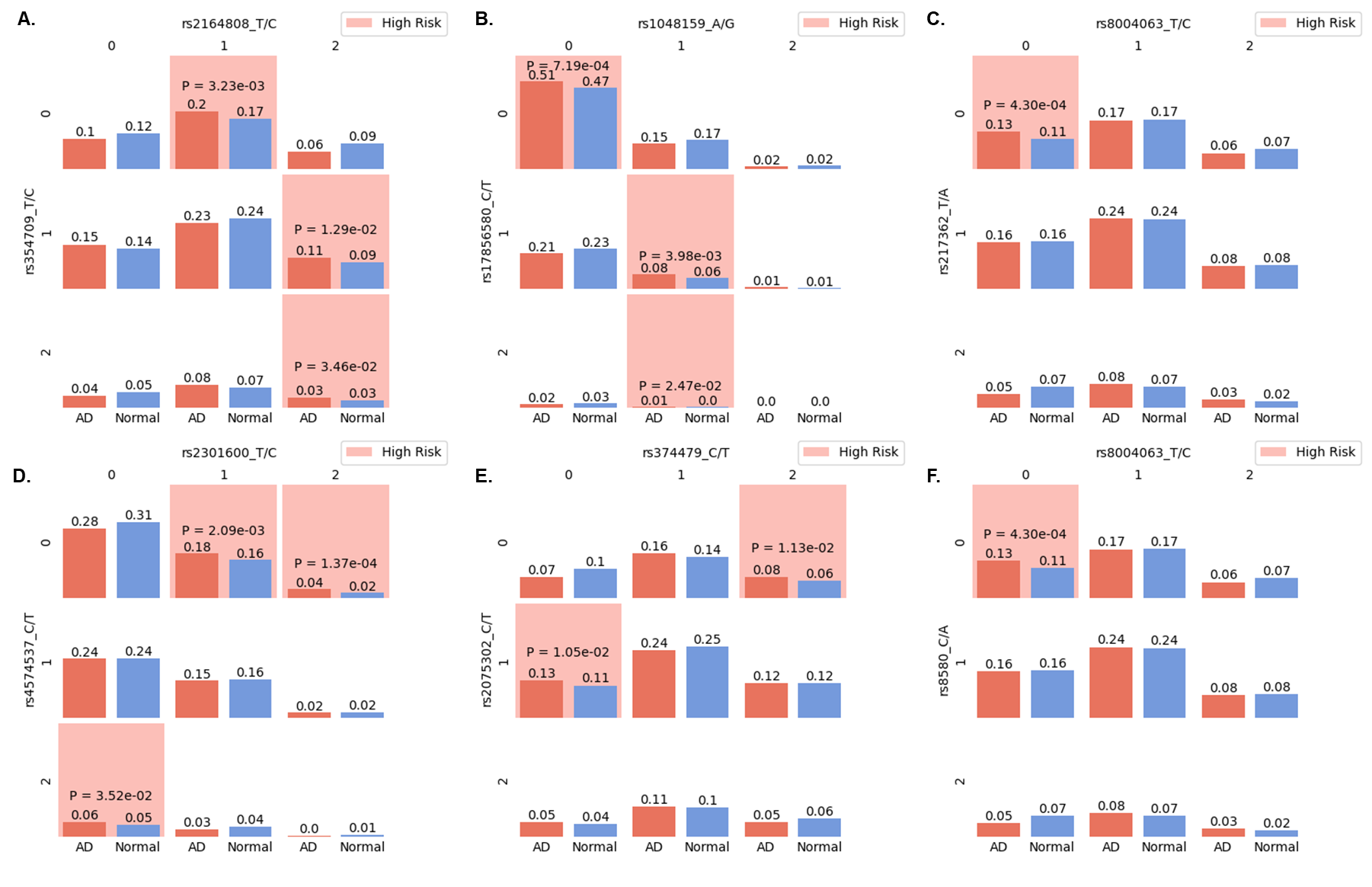


**Figure S2. Visualization of SNP-SNP interactions.** The ratio of case and control in each cell is shown. Cell with significant higher cases than controls by fisher’s exact test are marked red.

**
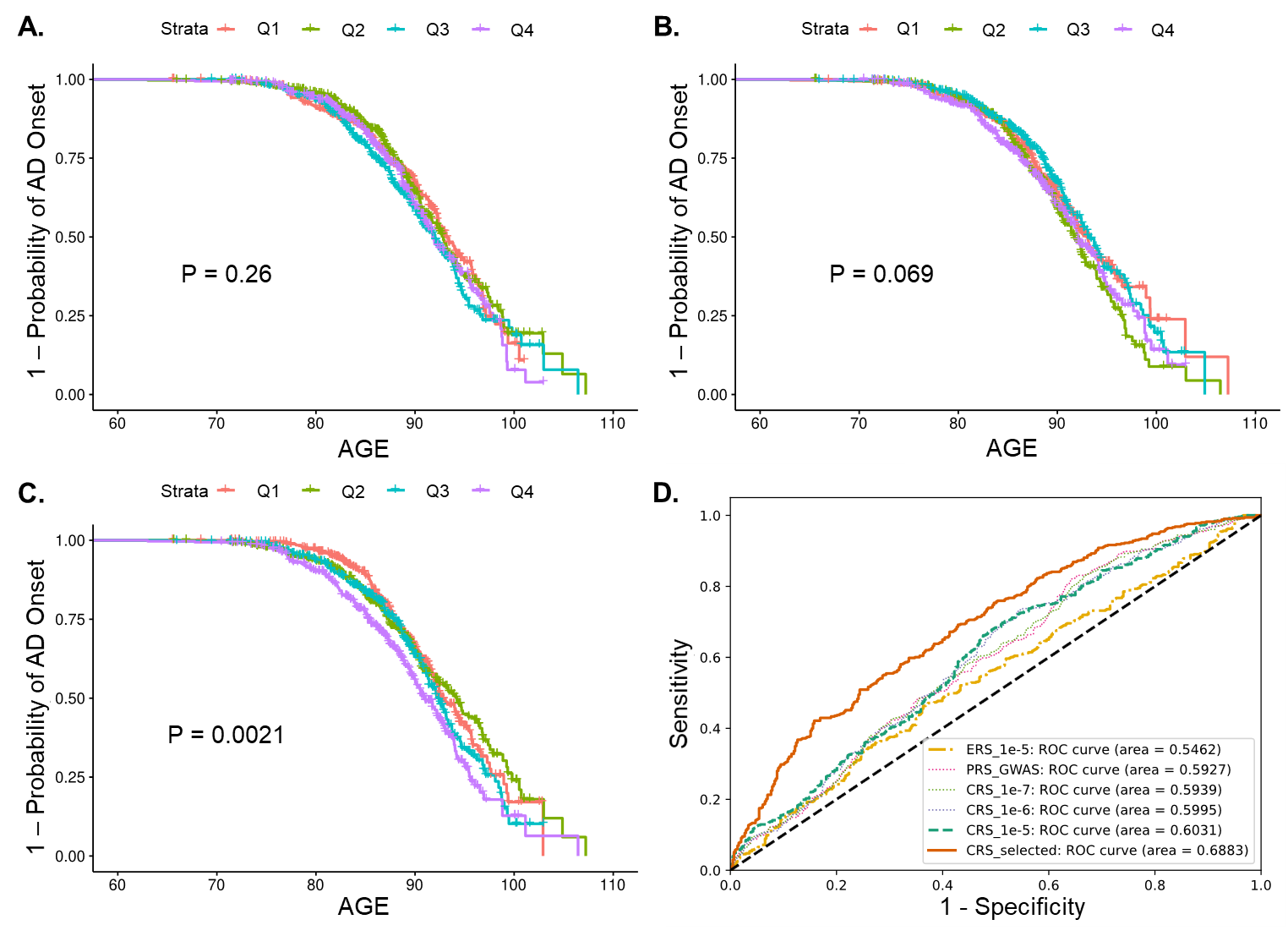
**

**Figure S3. Performance of epistasis risk scores (ERSs), polygenic risk scores (PRSs) and combined risk scores (CRSs) in AD risk prediction using samples from ROS/MAP.** Samples were divided into four quantiles (Q1 to Q4: from the lowest risk to the highest risk) based on their ERSs. The probability of developing AD was analyzed by the Kaplan-Meier method, where the *P* value was obtained by the log-rank test. ERSs were obtained via interactions with **(A)** *P* < 1 × 10^-7^, **(B)** *P* < 1 × 10^-6^, or **(C)** *P* < 1 × 10^-5^. **(D)** ERS_1e-5: ERSs constructed by genetic interactions with *P* value smaller than 1 × 10^-5^; PRS_GWAS: PRSs constructed by APOE (rs7412 and rs429358) and 20 SNPs identified by previous GWAS; CRS_1e-7, CRS_1e-6, CRS_1e-5: combined risk score of SNPs and SNP-SNP interactions with a *P* value smaller than 1 × 10^-7^, 1 × 10^-6^, or 1 × 10^-5^; CRS_selected: similar to CRS_1e-5, except that only genetic interactions showing non-random effects in ROS/MAP and ADNI were kept.


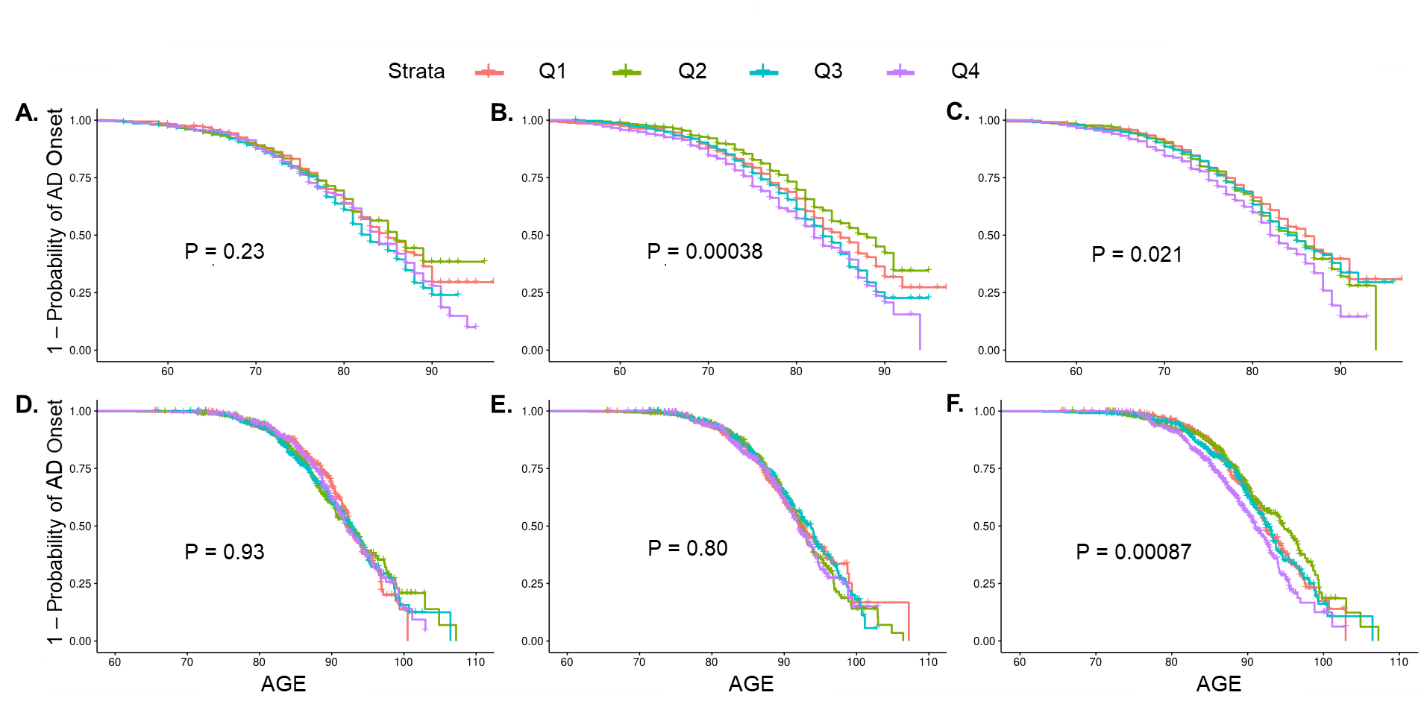


**Figure S4.** **Epistasis risk scores (ERSs) analysis after removing genetic interactions that showed main effects (*P* < 0.05) using samples from ADNI and ROS/MAP.** Samples are divided into four quantiles (Q1 to Q4: from the lowest risk to the highest risk) based on their ERSs. The probability of developing AD was analyzed by the Kaplan-Meier method, where the *P* value was obtained by the log-rank test. ERSs for individuals in ADNI were obtained via interactions with *P* < 1 × 10^-7^ **(A)**, *P* < 1 × 10^-6^ **(B),** or *P* < 1 × 10^-5^ **(C)**. ERSs for individuals in ROS/MAP were obtained via interactions with *P* < 1 × 10^-7^ **(D)**, *P* < 1 × 10^-6^, **(E)** or *P* < 1 × 10^-5^ **(F)**.


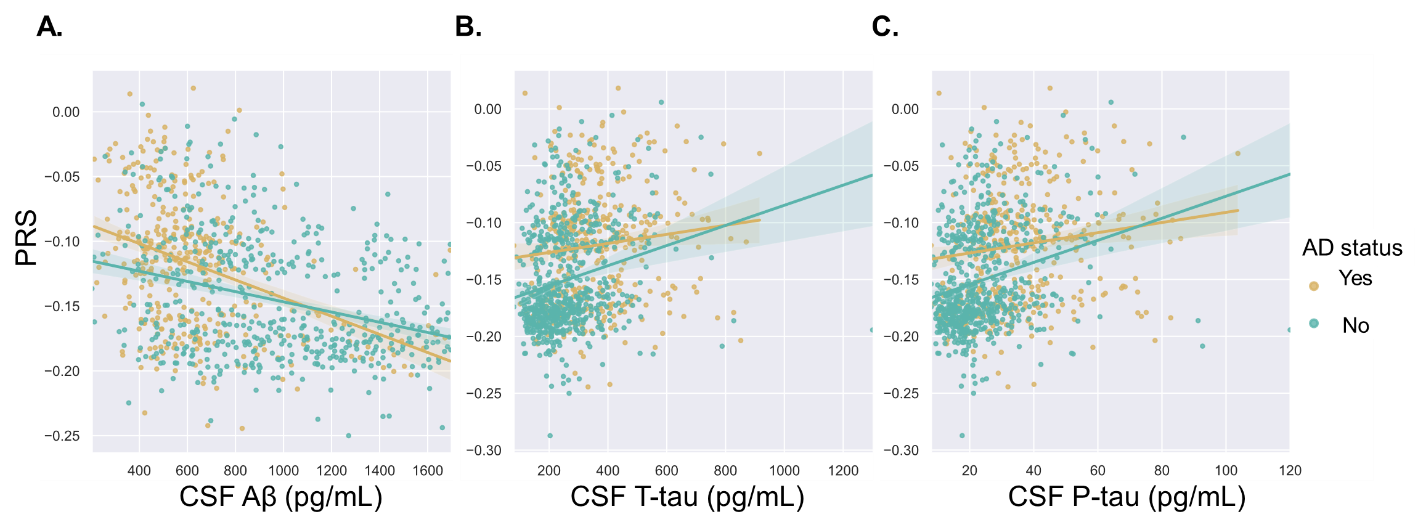


**Figure S5.** **Associations between PRSs (polygenic risk scores constructed by *APOE*, i.e., rs7412 and rs429358 and 20 SNPs identified by previous GWAS) and Alzheimer’s disease pathologies. (A)** PRSs are negatively correlated with CSF Aβ1-42 (AD (n = 388): R2 = -0.41, P = 3.0 × 10^-17^; non-AD (n = 655): R2 = -0.36, P = 3.4 × 10^-21^). **(B)** PRSs show a positive correlation with CSF total tau (AD (n = 388): R2 = 0.11, P = 0.027; non-AD (n = 655): R2 = 0.23, P = 4.8 × 10^-9^). **(C)** PRSs show a positive correlation with phosphorylated tau (AD (n = 388): R2 = 0.14, P = 0.0067; non-AD (n = 655): R2 = 0.26, P = 6.77 × 10^-12^).
